# Laminar organization of cellular microcircuits modulating human interictal epileptiform discharges

**DOI:** 10.1038/s41593-026-02258-4

**Published:** 2026-04-30

**Authors:** Alexander B. Silva, Siddharth A. Marathe, Quinn R. Greicius, Duo Xu, Shailee Jain, Jason E. Chung, Xiaofang Yang, Ankit N. Khambhati, Matthew K. Leonard, Jonathan K. Kleen, Edward F. Chang

**Affiliations:** 1 Department of Neurological Surgery, University of California, San Francisco, San Francisco, CA USA; 2 Weill Institute for Neuroscience, University of California, San Francisco, San Francisco, CA USA; 3 University of California, Berkeley, San Francisco Graduate Program in Bioengineering, Berkeley, CA USA; 4 Department of Neurology, University of California, San Francisco, San Francisco, CA USA

**Keywords:** Neuronal physiology, Epilepsy, Neural circuits, Perception, Neural decoding

## Abstract

Interictal epileptiform discharges (IEDs) are pathological bursts of brain activity between seizures in people with epilepsy. Despite their importance in diagnosis, cognitive comorbidities and therapeutic implications as biomarkers for neurostimulation, it is unknown how IEDs arise from structured large-scale neuronal firing across human cortical lamina. We used high-density Neuropixels probes to record from epileptogenic tissue in patients undergoing resective surgery, sampling 1,152 neurons during 1,094 IEDs across nine neocortical sites. We identified microcircuits for IEDs organized by firing pattern, neocortical depth and putative cell type. Regular-spiking cells, concentrated in superficial cortical lamina, initiated and coded the amplitude of the sharp discharge and excitatory–inhibitory imbalances across neocortical lamina preceded IEDs, enabling IED prediction up to 1,000 ms in advance. Most neurons that were modulated during IEDs also encoded cognitive information and adhered to physiological rhythms at baseline. Thus, neocortical IEDs are generated from predictable laminar–cellular interactions, providing the groundwork for new neurostimulation therapies that harness the granularity of single neurons.

## Abstract (web summary)

High-density single-neuron recordings in patients with epilepsy revealed interictal discharges are generated by structured laminar circuits. These circuits overlapped with cognitive circuits and could predict discharges up to 1 s in advance.

## Main

Epilepsy is a common neurological condition defined by seizures (episodes of abnormal electrical activity in the brain) affecting over 50 million people worldwide^1^. Between seizures, abnormal neural circuits emit brief pathological bursts of electrical activity, termed interictal epileptiform discharges (IEDs)^2^. IEDs are helpful for localizing epileptogenic tissue^3^ but may themselves contribute substantial cognitive comorbidities for patients with epilepsy, even when seizures are controlled^4,5^. Further, cyclical fluctuations in IED rate have been shown to forecast periods of heightened seizure risk, along with other emerging clinical applications^6,7^.

Despite their importance, whether IEDs are born from random synchronous neuronal firing in the human cortex, or from structured firing across laminar microcircuits (predictable phases and neuronal constituents), is generally unknown. Mechanistic knowledge could guide new strategies in suppressing epileptiform activity and reorganizing pathological networks through plasticity. Yet, such knowledge requires large-scale recordings from numerous neurons across the entire cortical depth, which have not been feasible in vivo in humans. Thus, there is strong clinical motivation to apply new technologies to understand the laminar structure of cellular microcircuits that give rise to IEDs, as this would lead to new clinical applications in the near-term future.

A particularly relevant clinical application of IEDs is closed-loop responsive neurostimulation (RNS)^8^. RNS requires several years of therapy to demonstrate optimal efficacy^9^, as its main mechanism is thought to be modulation of network excitability, which produces slow changes in plasticity over time^10^. Each day, a typical patient with RNS receives thousands of electrical brain stimulations in the seizure onset zone/network (SOZ) in response to IEDs (more rarely to actual seizure onsets). There is a desperate need to accelerate this treatment effect and to improve its efficacy: a median 75% reduction in seizures is observed only after 2–9 years of therapy and less than 20% of patients achieve seizure freedom^9,11^. Of note, these therapies are currently limited to delivering stimulation after IEDs are detected and evident on macroelectrode recordings (‘responsive’ to IEDs that have already come to fruition). However, stimulation during or before IED waveforms might have greater therapeutic efficacy by disorganizing IEDs, or even preventing their formation^12,13^. Such goals would require a mechanistic understanding and identification of local microcircuit signatures that predict IEDs with sufficient lead times.

How might firing of individual neurons, across different cell types and neocortical depths, form microcircuits for generating IEDs? Previous studies in animal models have proposed a paroxysmal depolarization mechanism of IED generation, in which a synchronous burst of numerous neurons is followed by a period of relative inhibition^14–16^. Under this model, modulations in neuronal firing are closely aligned to the IED deflection; however, a study of human IEDs with microelectrodes identified more heterogeneous neuronal firing patterns that were not necessarily predicted by a paroxysmal depolarization model^17^. Thus, there is reason to believe human IEDs may leverage a more complex neuronal microcircuit, potentially spanning a network of distinct cell types and firing patterns. Previous work suggests that such microcircuits may be driven by excitatory–inhibitory imbalance^18–21^ and organized across neocortical layers^22–24^. Further, previous macroelectrode studies^25–28^ have correlated IEDs with transient cognitive impairment (TCI). The degree to which IEDs modulate hippocampal neurons has been correlated with impaired confidence during memory retrieval^29^, suggesting microcircuits for IEDs may overlap to some degree with neurons important for cognition, forming a potential mechanism of TCI.

To test whether IEDs emerge from predictable and coordinated laminar microcircuits, we recorded single-neuron firing (up to 189 simultaneously recorded neurons per site) with high-density Neuropixels (NP) probes^30^ during IEDs in patients undergoing awake resective surgery for seizures. Leveraging high-throughput sampling, we identified laminar–cellular IED microcircuits organized by firing pattern, putative cell type and cortical depth. At baseline, neurons modulated during IEDs also encoded cognitive information and adhered to physiological rhythms. Spiking patterns of large-scale neuronal ensembles within distinct cortical insertion sites could predict not only IEDs, but also their pathological features such as amplitude and propensity to form a series of discharges, up to a second before their onset, suggesting the feasibility of future antecedent neurostimulation therapies in epilepsy^31–34^.

## Results

### Neuropixels recordings and IED processing

**Fig. 1:**
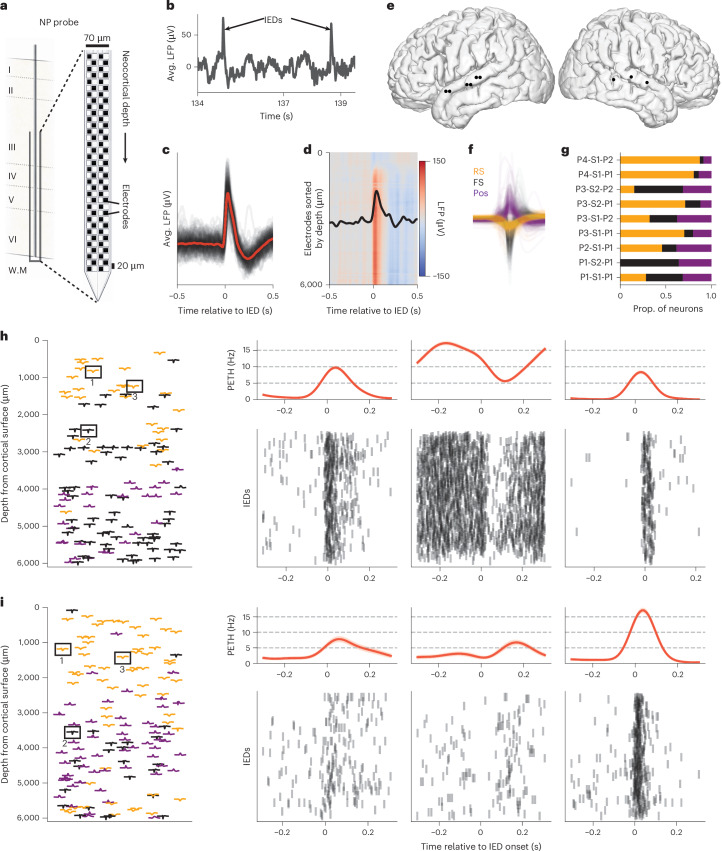
Neocortical neuron firing rates across depth are modulated immediately before, during and after local IEDs. **a**, Schematic of the NP probe, with an approximation of extent across neocortical laminar depth, based on previous work^30^. **b**, Example LFP trace averaged across NP electrode contacts showing IEDs. **c**, All IEDs were isolated from an example patient, mean across IEDs in red. IEDs were aligned to the maximum absolute slope. **d**, LFP across all NP channels during a single IED. **e**, Recording sites in this study (black dots). Directly adjacent dots reflect the use of multiple probes to measure distinct cortical insertion sites during the same recording period. **f**, Waveforms of isolated neurons, across all NP probe insertions. Color reflects the waveform type (FS, fast-spiking; RS, regular-spiking; Pos, positive-spiking), identified with clustering. **g**, Breakdown of waveform type by insertion site (P, patient; S, site; P, probe; note that the first ‘P’ indicates patient, whereas the last refers to probe number for that patient). Nine cortical sites (probe insertions) were sampled, relative to six distinct recording sites, due to three sites having a double-probe apparatus (**a** and Methods, ‘Isolation of IEDs’). **h**,**i**, Two sample cortical insertion sites from different patients. Each neuron is plotted by depth and colored by waveform type (left). Three example neuron (boxes in left) rasters of firing aligned to IED maximal absolute slope, visualized across IEDs (rows) (right). For each neuron, a smoothed peri-event time histogram (PETH; 100-ms bins, 10-ms step size; red envelope shows s.e.m.) is shown. Distinct single-neuron firing and waveform patterns are seen across neocortical depth. Activation around IED onset is seen in example regular-spiking neurons, whereas suppression around IED onset is seen in example fast-spiking neurons. Neurons with distinct peri-IED response patterns exist within small distances over neocortical depth. Across all figures and panels, shaded error bars reflect s.e.m. Source data

**Extended Data Fig. 1:**
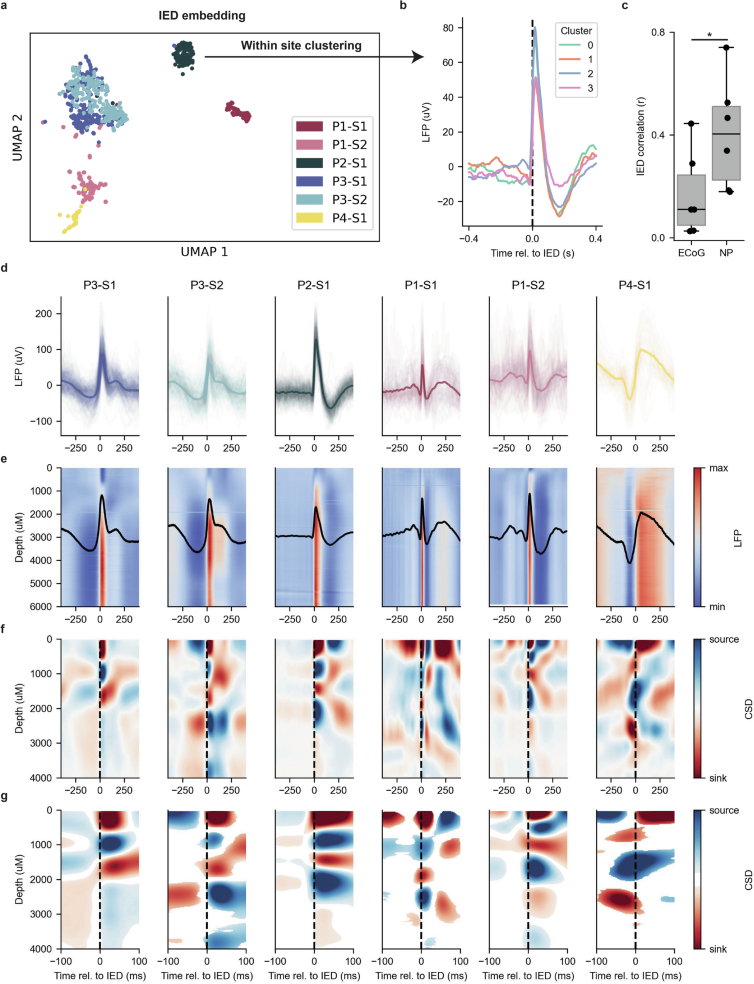
Characteristics of IED LFP waveforms across recording sites. (**a**) Waveforms of IEDs (average across NP channels and aligned to maximal slope) underwent low-dimensional embedding using uniform manifold approximation and projection (UMAP) and projected into 2D space (UMAP dimension 1 vs. 2) to assess waveform heterogeneity. For dual-probe recordings, LFP data from only one probe is included in light of minimal overt waveform differences. **(b)** K-means clustering (n = 4, elbow method) reveals minimal heterogeneity of IEDs within a single site with most variance in the maximal amplitude. (**c**) For each site we computed the mean cross-correlation between IEDs. We did this for NP recordings and preoperative electrocorticography (ECoG) recordings. Higher overall correlation is seen between IEDs captured on NP recordings than ECoG (P = 0.041; U = 5; 6 ECoG sites and 6 NP sites; Two-sided Mann-Whitney U test test). **(d)** Raw waveforms of IEDs, colored the same as **(a)** for each NP recording site. **(e)** Average IED waveform across cortical depth for each NP recording site. Note the polarity flip in the very most superficial channels. **(f)** Current source density averaged across IEDs for each recording site. **(g)** Current source density in the immediate window around the IED-maximal slope (± 100 ms). Pixels that were below the 99.5th percentile of a null distribution, by aligning to random times within the block (see Methods) are set to 0 (white). Source data

To understand how human single neurons, distributed across neocortical depth, give rise to IEDs, we leveraged NP probes to record from cortical lamina during awake brain surgeries for drug-resistant epilepsy^35,36^. Probes were placed in tissue that was resected as part of surgical treatment in people with epilepsy/seizures. Technical protocols and hardware were optimized to record from the brain’s lateral neocortex. Each probe had 384 active electrodes inserted trans-laminarly across the neocortical depths and spanning a range of 7.66 mm along the shank (Fig. 1a). Local field potential (LFP) recordings from these same electrodes enabled identification of IEDs using a semi-automated detection algorithm followed by manual verification (Fig. 1b and Methods). In general, IEDs had similar waveform profiles among contacts spanning the neocortical depth (Fig. 1c) and within patients (Fig. 1d and Extended Data Fig. 1).

**Extended Data Fig. 2:**
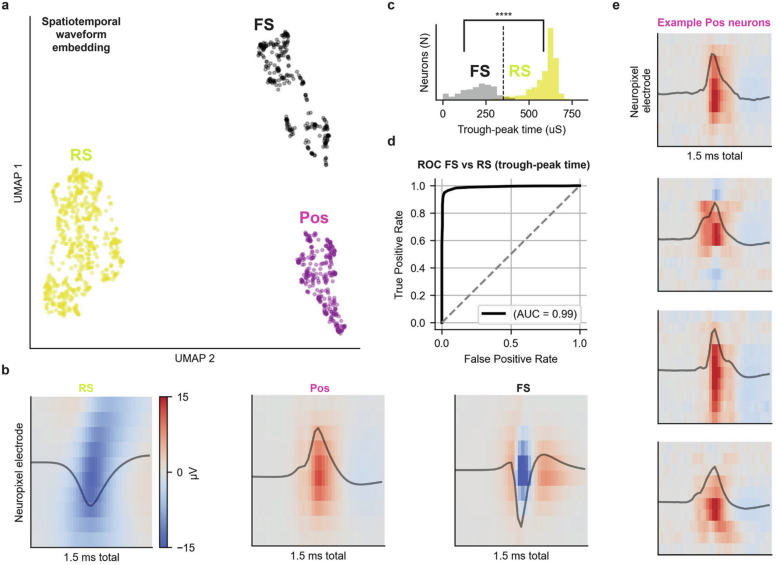
Unsupervised clustering of spatiotemporal neuronal spike waveforms. (**a**) Uniform Manifold Approximation and Projection (UMAP) embedding of each neuron’s spatiotemporal spike waveform (10 closest channels; 1.5 ms total time). Clusters were defined with a simple gaussian linear mixture model and are clearly visible by eye. (**b**) Average spatiotemporal waveform for RS, FS, and Pos spiking neurons. (**c**) Distribution of Trough-peak time across FS and RS neurons (P = 1.2e-124; U = 166333; 587 RS and 279 FS; Two-sided Mann-Whitney U test test). Dotted line is at 350 uS (**d**) Receiver operating curve for separating FS from RS neurons using the trough-peak time. Note that the total area under the ROC curve (AUROC) is 0.99. (**e**) Example spatiotemporal waveforms from 4 positive spiking neurons. Source data

We screened 24 NP recordings across 11 patients, inherently selecting for patients with epileptogenic zones that included lateral temporal neocortex due to the above constraints. All recordings took place in tissue clinically determined to be in the epileptogenic zone, which was subsequently resected (Supplementary Table 1). We identified nine distinct NP insertion sites across four patients who showed occasional to frequent IEDs (71–309 IEDs per site; Supplementary Table 1). We isolated individual neurons from each of these recordings using automated and manual spike sorting (Methods). For each neuron, we extracted the spatiotemporal spike waveform, on the ten closest electrodes, and clustered spatiotemporal waveforms across neurons, finding three waveform types (Methods and Extended Data Fig. 2) that align with previous work: fast-spiking, regular-spiking and positive-spiking^35,37–39^. Previous work has demonstrated that the most salient differentiating metric between regular-spiking and fast-spiking neurons, both having negative spike deflections, is the trough-peak time on the best isolated channel^35,40,41^ (Fig. 1f). Indeed, in our data, regular-spiking neurons had significantly longer trough-peak times (Extended Data Fig. 2), with regular-spiking and fast-spiking neurons separable with 0.99 area under the receiver operating curve (AUROC) using trough-peak time (Extended Data Fig. 2). The optimal cutoff trough-peak time was around 350 μS, similar to previously reported work^35,41^. Recordings provided 520 s to 1,380 s of continuous artifact-free recording periods and yielded between 14 to 189 neurons each (Fig. 1g and Supplementary Table 1).

We aligned neuronal spiking within recording blocks to the maximal absolute slope of IEDs (sharpest edge), a temporally specific pathophysiological feature^42,43^. Neuronal spike waveforms and depth relative to the cortical surface from two different patients are shown in Fig. 1h,i (left). Current source density (CSD) analyses revealed significant sink–source pairs in each distinct recording site (*P* < 0.01; permutation test). LFP polarity reversals and multiple source–sink pairs that extended to deeper cortical layers (Extended Data Fig. 1) were generally consistent across patients, supportive of locally generated rather than propagated IEDs^22,23^ though simultaneous macroelectrode recording of surrounding structures was not available.

### Single-neuron activity during IEDs

**Fig. 2:**
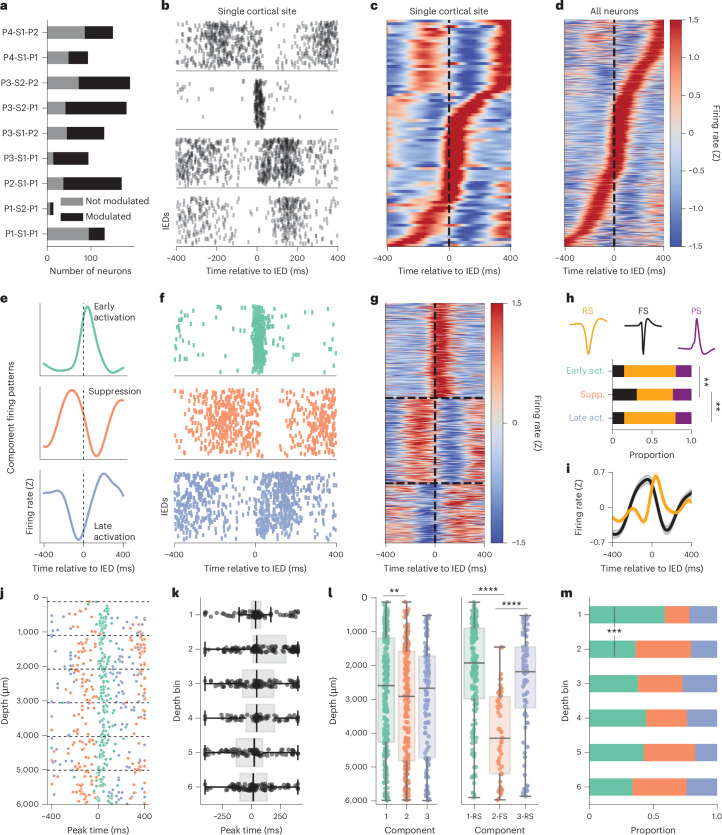
Peri-IED firing patterns of single neurons across neocortical depth. **a**, Proportion of neurons modulated by IEDs in each insertion site (P, patient; S, site; P, probe; as in Fig. 1g). **b**, Temporal variability in single-neuron responses seen within a cortical site. **c**,**d**, Normalized PETH of all neurons modulated by IEDs in the cortical site shown in **b** (**c**) and all neurons across sites (**d**). **e**, Three temporal firing patterns (early-activation, suppression, and late-activation activity) found with unsupervised clustering across all modulated neurons with *k*-means. **f**, Sample IED-aligned rasters for an example neuron showing each firing pattern. **g**, PETH of all modulated neurons sorted by firing pattern. **h**, Proportion of neurons with each firing pattern having fast-spiking (FS), regular-spiking (RS) or positive-spiking (PS) waveform types (as in Fig. 1g; **P < 0.01; two-sided permutation test over *n* = 2,000 iterations). **i**, Firing rate averaged across all FS and RS neurons. **j**, Neurons with peak firing times span the peri-IED interval and neocortical depth (color: firing pattern). **k**, Distributions of peak firing times across binned neocortical depths (*P* > 0.05; 83, 122, 103, 68, 87 and 92 neurons; pairwise two-sided Wilcoxon rank-sum tests with Holm Bonferroni correction). **l**, Distribution of the three firing patterns across depth (**P = 0.01; *Z* = 2.4; *n* = 230 and 201 neurons; two-sided Wilcoxon rank-sum tests) (left) and based on putative cell types (****P = 5.4 × 10^−14^, ****P = 5.8 × 10^−9^; *Z* = 7.5, 5.8; 158, 58 and 84 neurons; two-sided Wilcoxon rank-sum tests) (right). **m**, Proportion of each firing pattern based on depth bin (***P = 1.1 × 10^−3^; *Z* = 3.2; two-sided proportion *z*-test component 1, bin 1 versus 2). Boxplots across all figures and panels depict median (horizontal line inside box), 25th and 75th percentiles (box), 25th and 75th percentiles ±1.5× interquartile range (whiskers) and outliers (dots). Source data

**Extended Data Fig. 3:**
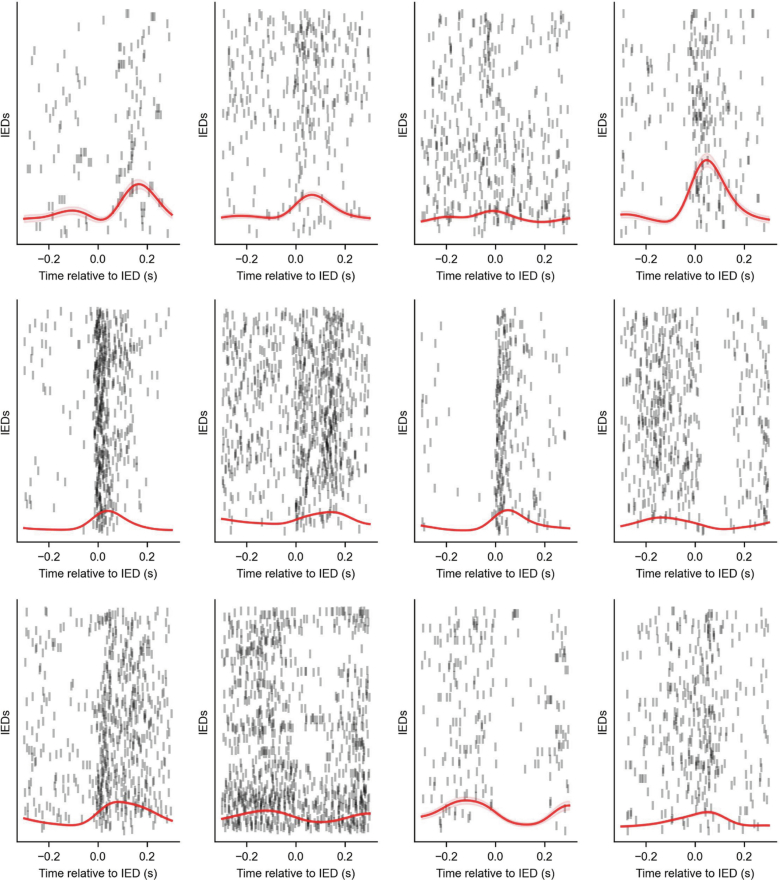
Additional examples of single-neuron firing aligned to IEDs. As in Fig. 1h-i, spiking is aligned to IED-maximal absolute slope. Here, the smoothed perievent time histogram (PETH), calculated as Fig. 1h-i, is overlaid on the raster in red (envelope: SEM). Overall, we observe substantial temporal heterogeneity in the response patterns of neurons during IEDs. Source data

Following previous work^17,29^, we evaluated a peri-IED interval of ±400 ms around the maximal IED slope. Temporal heterogeneity in neuronal responses was evident in raw rasters (Fig. 1i,j right, and Extended Data Fig. 3). Thus, to identify neurons that had modulations in firing associated with IEDs, we tested whether each neuron’s maximal firing rate was different from its minimal firing rate across IEDs during the peri-IED interval (Wilcoxon signed-rank test; false discovery rate (FDR)-corrected at α = 0.05). Across cortical insertion sites, 22.6–85.1% of neurons were modulated during IEDs (Fig. 2a–c). Firing rates across neurons were asynchronous and tiled the peri-IED interval, evident in single insertion sites (Fig. 2c) and when aggregated across all neurons (Fig. 2d), and in contrast to paroxysmal depolarization models of IEDs^14–16^. It instead suggests that IEDs are generated by a complex microcircuit of cells, with subsets of them maximally modulated during different phases of the IED (before, during or after).

### Cellular–laminar microcircuits for IEDs

**Extended Data Fig. 4:**
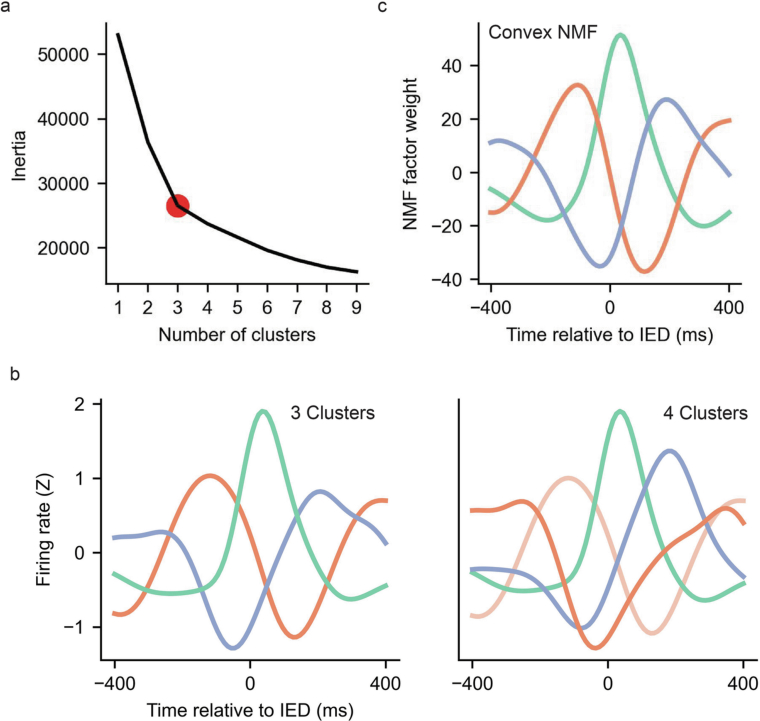
Number of firing rate clusters using K-means algorithm. (**a**) Distance from data-points to their assigned cluster centroid (inertia) as a function of number of K-means clusters. The optimal number of clusters based on the elbow method is annotated. (**b**) To further support the use of 3 clusters, we compared cluster templates with using 3 vs. 4 clusters. The addition of a fourth cluster (right panel) did not identify a unique temporal pattern, rather the suppression cluster (orange) appeared split into redundant clusters with a minor temporal shift of the peak. (**c**) Convex non-negative matrix factorization (NMF), a soft clustering technique, corroborates the same three firing pattern components as K-means, a hard clustering technique. Source data

To better define microcircuits and the role of various cell types across neocortical depth for IED generation, we clustered the IED-locked average firing rate patterns of the individual neurons using *k*-means (Methods and Extended Data Fig. 4; note that this is distinct from the separate uniform manifold approximation and projection (UMAP) clustering performed on the IED LFPs in Extended Data Fig. 1). We found three specific patterns of neuronal firing (Fig. 2e) that spanned the peri-IED interval.

1. ‘Early-activation’ pattern, with increased firing within 50–100 ms around the IED maximal slope, peaking just after it.
2. ‘Suppressed’ pattern, with an antecedent increase in firing and rapid decrease around IED maximal slope, possibly due to depolarization block^44^.
3. ‘Late-activation’ pattern, with an antecedent decrease 0–200 ms before the IED followed by increased firing 100–300 ms after the IED maximal slope.

Examples of these types, in the same NP insertion, are shown in Fig. 2f, and sorted by cluster across all neurons in Fig. 2g. Components 1 and 3 were dominated by regular-spiking cell types, whereas the suppression component (2) had relatively more fast-spiking cells (putative inhibitory neurons)^45–47^ (Fig. 2h; *P* < 0.01; two-sided permutation test; Methods). These relations were reflected when averaging firing rate patterns across fast-spiking and regular-spiking cells (Fig. 2i), grounding our firing rate components in partially distinct cellular subtype proportions.

We next studied how this circuit manifested across neocortical depth^22,23^. Neurons across the neocortical depth showed varying peak activation times from −300–300 ms relative to IED maximal slope (Fig. 2j). Wide variance in peak timing was evident when discretizing depth^39^ into six equal bins with no difference in median peak time across bins (Fig. 2k; *P* > 0.05, two-sided pairwise Wilcoxon rank-sum tests with Holm Bonferroni correction). Correspondingly, each of the three neuronal firing patterns was found across cortical depth (Fig. 2l); however, early-activation neurons (component 1) were more concentrated to superficial depths (Fig. 2j–m; *P* < 0.01; two-sided Wilcoxon rank-sum test) and this became more apparent when subgrouping the two activation components (1 and 3) to putative excitatory neurons (RS waveform) and the suppression component to putative inhibitory neurons (fast-spiking waveform; Fig. 2l; *P* < 0.0001; two-sided Wilcoxon rank-sum tests). This is consistent with superficial cortical layers being key for initiation of the sharp discharge^22,23^. Although, our results most strongly highlight that single-neuron firing patterns are more variable and distributed across the neocortical depth than previously assumed. This aligns with the presence of multiple source–sink pairs across cortical depth, as defined with CSD analysis (Extended Data Fig. 1).

**Fig. 3:**
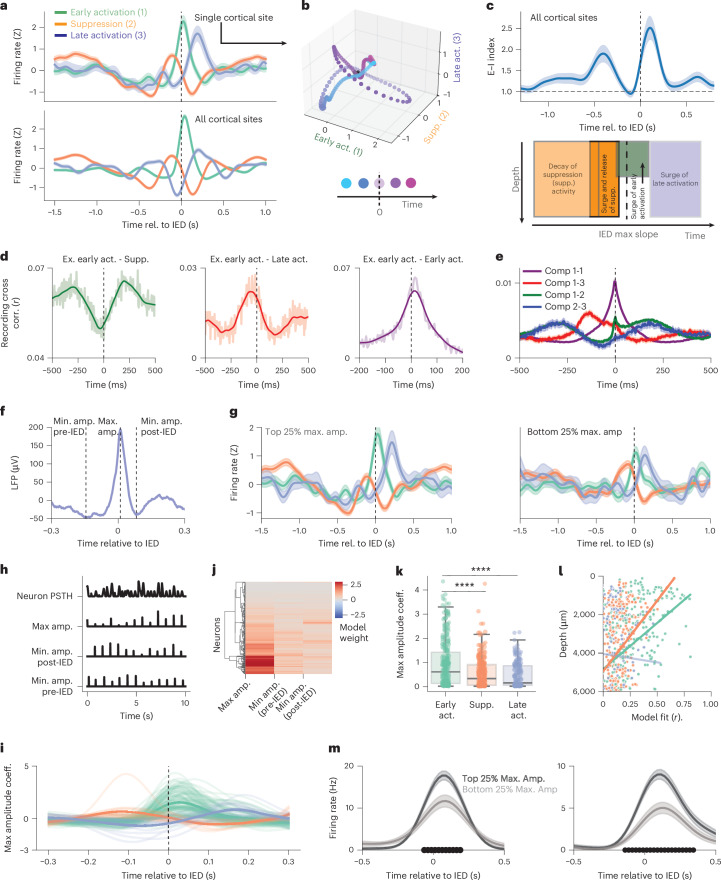
Cellular–laminar microcircuits for IED generation based on excitatory–inhibitory dynamics. **a**, Average activity of early-activation, late-activation and suppression neurons (as in Fig. 2e) leading up to an IED. **b**, Visualization of activity across the three populations as a trajectory in three-dimensional space over time. **c**, Top: computed excitatory–inhibitory (E–I) index (Methods). Bottom: model of IED generation from the circuit based on **a**,**b**. **d**, Cross-correlation functions between recording-wise spike trains of example neurons **e**, Average cross-correlation functions between neurons belonging to each of the components (1, early-activation; 2, suppression; 3, late-activation; cross-correlation permutation test; Methods). **f**, Example IED with key features annotated. **g**, Average firing of neurons from each component (colors as in **a**) for high- and low-amplitude IEDs. **h**, Time-lagged linear model used to predict neuronal firing from features of the IED. **i**, The non-time collapsed maximum amplitude coefficient weights for neurons with significant fit (model *r*^2^ > 0.05). **j**, For each neuron (hierarchically clustered), the maximum coefficient for each IED feature is visualized. **k**, Early-activation neurons had the strongest coefficient strength for the maximum amplitude (****P = 2.2 × 10^−6^, ****P = 8.4 × 10^−9^; *Z* = 4.7, 5.8; 279, 251 and 175 neurons; two-sided Wilcoxon rank-sum tests). **l**, Model fit (*r*) as a function of neocortical depth, demonstrating better fits more superficially (*P* = 1.6 × 10^−13^, *P* = 2.4 × 10^−8^, *P* = 0.31; *r* = −0.42, −0.34, 0.08; 279, 251 and 175 early-activation, suppression and late-activation neurons; two-sided Spearman correlation). **m**, Mean PETH for two example early-activation neurons. Dots indicate significantly different time points (*P* < 0.05; two-sided cluster permutation test with multiple comparison correction). Source data

To understand how excitatory–inhibitory imbalances (for example decay in inhibition^18^) at the neuronal level may contribute to IED generation as suggested previously^18–20,48^, we computed the IED-aligned average firing rate patterns of the three neuron component groups (early activation, suppression and late activation) as a three-dimensional dynamic trajectory (Fig. 3a,b). Up to 1,000 ms before the IED, firing of suppression neurons decreased (Fig. 3a,b). This was followed by a brief surge and release of suppression activity, with the release corresponding to surging activity in early-activation (timed to the IED discharge) and then late-activation neurons. This was followed by restoration of suppression activity in the immediate post-IED interval. To more explicitly quantify excitatory–inhibitory imbalance, we defined a modified excitatory–inhibitory index as the activity in early and late-activation neurons divided by the activity in suppression neurons. Matching previous results, the excitatory–inhibitory index gradually increases from baseline before the IED. IED initiation is marked by a drop then strong increase in the excitatory–inhibitory index, followed by a return to baseline (Fig. 3c). Overall, different neuronal subtypes (1) contribute to excitatory–inhibitory imbalance, (2) are important during different phases of the IED and (3) are modulated hundreds of milliseconds before IED maximal slopes (Fig. 3c).

**Extended Data Fig. 5:**
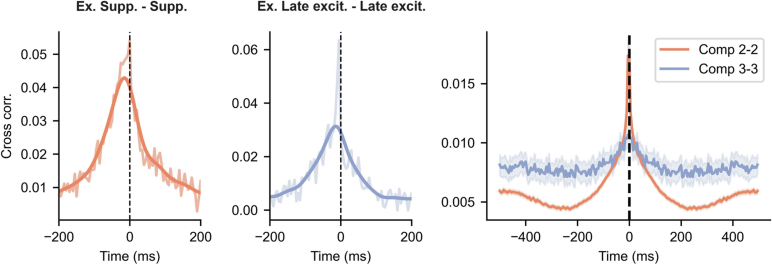
Cross-correlations within suppression and late excitatory neurons. Example cross correlograms between neurons within the suppression and late excitatory component firing patterns. Average across all pairs is shown on the right and mirrors early excitatory neurons (Fig. 3d-e). Shaded error bars reflect standard error of the mean. Source data

**Extended Data Fig. 6:**
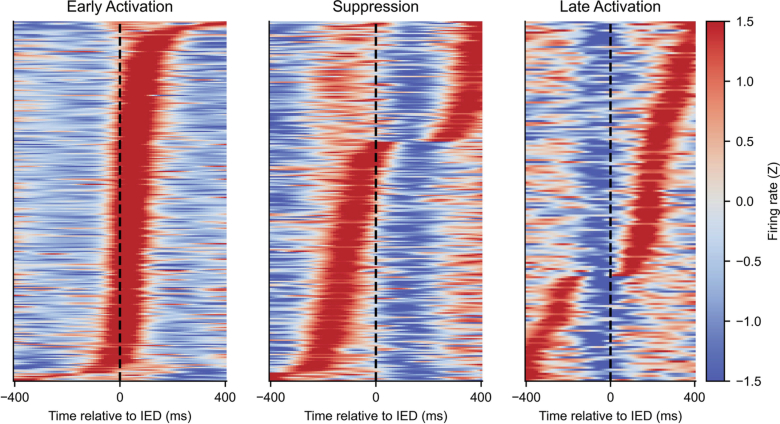
Asynchronous neuronal firing within activation pattern components. For neurons clustered to each component firing pattern relative to IEDs (Fig. 2e), firing rates are sorted by peak activation time. Variance in peak activation time is visible across the early, activation, suppression, and late-activation components. This provides further evidence that even within component firing patterns, activations are asynchronous. Source data

We further analyzed differences in peri-IED timing by evaluating neuronal connectivity within the microcircuit^49^. To complement our previous analyses, we used neuronal spike trains across entire recording sessions (not just peri-IED intervals) as well as a finer time resolution of 5-ms bins. We computed pairwise cross-correlations between the strongest modulated neurons in each firing rate component within cortical insertion sites (Fig. 2a shows modulated neurons surpassing *P* < 0.0001; Methods shows how significance of each pairwise cross-correlation was assessed). Early-activation neurons had peaks in firing both before and after inhibitory neurons, but were not highly correlated at zero lag (Fig. 3d,e). In addition, they fired before late-activation neurons with lower correlation at no lag. Notably, there were significant nonzero lagged cross-correlations within neurons of each component firing pattern (Fig. 3d,e and Extended Data Fig. 5). This demonstrates intrinsic connectivity between neurons of the IED generation circuit and further highlights asynchronous firing even within neuronal firing components (Extended Data Fig. 6).

**Extended Data Fig. 7:**
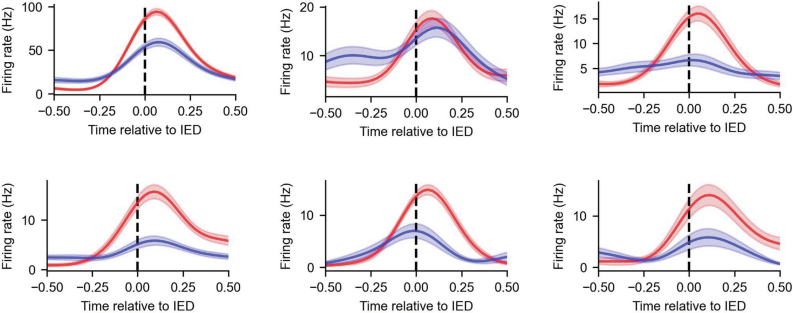
Additional examples of neurons coding IED amplitude. Mean PETH for example neurons with modulated firing during higher amplitude IEDs (Red: top quartile of IED amplitudes, Blue: bottom quartile). Shaded error bars represent standard error of the mean. Source data

As IEDs are emergent LFP events generated by microcircuits, we evaluated whether individual neurons could predict key pathological IED features (Fig. 3f) such as peak amplitude^50^, which is correlated with the degree of IED-associated cognitive impairment^51^. Motivating this analysis, comparison of IEDs in the highest and lowest amplitude quartiles showed that the ‘activation’ components (1 and 3) were attenuated while the inhibitory component (2) showed the reverse effect (Fig. 3g; all *P* < 0.05; Wilcoxon rank-sums tests). To evaluate for IED amplitude coding by individual neurons within the circuit, we fit a time-lagged linear model to predict single-unit firing rates from the IED amplitude as well as two control features: the minimum amplitude before and after the discharge (Fig. 3h). Many IED-modulated neurons (32.9%) exceeded a model *r*^2^ of 0.05 and the peak of the maximum amplitude coefficient over time corresponded with component firing pattern (Fig. 3i). Indeed, in terms of the strongest feature over time, the majority of neurons that significantly fit the model were most tuned to maximum amplitude (57.7%; Fig. 3j) and early-activation (component 1) neurons showed strongest relation between firing rate and higher amplitude IEDs (Fig. 3k; *P* < 0.0001; Wilcoxon rank-sums tests). In line with an enrichment of early-activation neurons in the superficial cortex, overall model fits were stronger at superficial neocortical depths (Fig. 3l; early-activation: *P* < 0.0001, *r* = −0.42; suppression: *P* < 0.0001, *r* = −0.34; late-activation: *P* > 0.05, *r* = 0.08; Spearman correlation of model *r* and depth). Example early-activation neurons showed clear differences in firing rate differences during highest and lowest quartile IEDs (Fig. 3m; also Extended Data Fig. 7 for additional example neurons).

**Extended Data Fig. 8:**
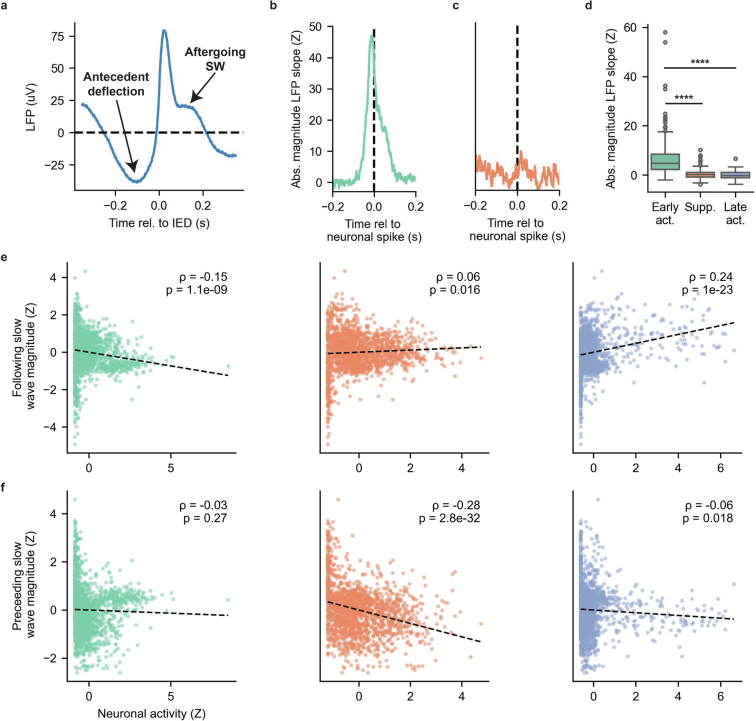
Coding of fast and slow components of IEDs. **(a)** Example IED from a single participant with the magnitude of the antecedent slow deflection and aftergoing slow wave labeled. LFP-absolute slope triggered average for an example early activation (**b**) and suppression neuron (**c**). (**d**) Distribution of LFP-absolute slope triggered magnitude (t = 0 in **b**) across all early activation, suppression, and late-activation neurons (P = 4.1e-52, 1.6e-47; U = 61755, 44118; 279 early-act, 251 supp, and 175 late act neurons; Two-sided Mann-Whitney U test tests). (**e**) Magnitude of the aftergoing slow wave as a function of firing rate across early activation, suppression, and late-activation neurons. (**f**) Same as (**e**) for the antecedent slow deflection (**e**-**f**, Two-sided Pearson Correlation) Source data

Finally, we directly asked how the three functional groups of neurons, early activation, suppression and late activation, give rise to slow and fast changes in the IED. IEDs are known to be composed of a fast core deflection followed by an aftergoing slow wave^50,52^. This aftergoing slow wave has been associated with the pathological extent of IEDs^27,53,54^. We first measured how sensitive spikes in each group of neurons were to fast or slow changes in the LFP. To do this, we averaged the instantaneous rate of change (absolute slope) of the LFP aligned to each neuron’s spikes. This ‘LFP slope’ triggered average then represents the rate at which the LFP is changing when a given neuron spikes. Overall, we found that early-activation neurons had a significantly higher LFP slope triggered average than suppression or late-activation neurons, associating the early-activation neurons with fast changes in the LFP and aligning with their role in coding the amplitude of the discharge (Extended Data Fig. 8). We next tested whether the relatively lower LFP slope triggered average in suppression and early activation neurons translated to a role in coding the amplitude of the antecedent and aftergoing slow wave. To do this, we computed the magnitude of the antecedent slow deflection and aftergoing slow wave for each individual IED. For each individual IED, we then calculated the mean firing rate across the three functional groups of neurons. Activity of late-activation neurons was positively correlated with the magnitude of the aftergoing slow wave, whereas activity in suppression neurons was negatively correlated with the antecedent slow discharge magnitude (Extended Data Fig. 8). Thus, these results establish that the properties of neuronal groups in the IED generation circuit (Fig. 3) support coding of slow changes by suppression and late-activation neurons.

### Neurocognitive correlates of IED generation circuit

**Fig. 4:**
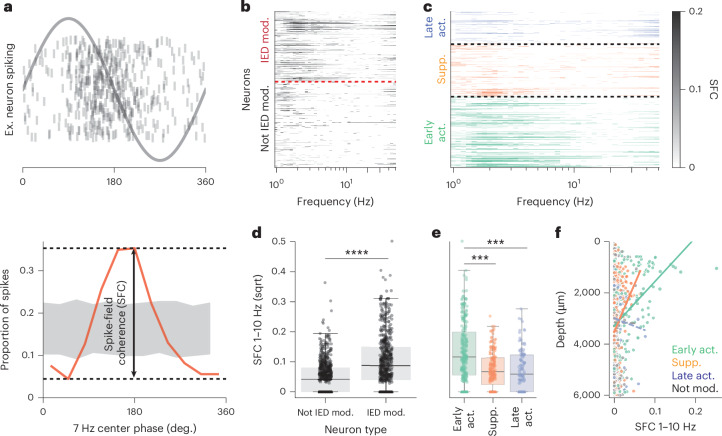
Spike-field coherence of IED microcircuits. **a**, Example neuron demonstrating neuronal spiking preference for 180° phase of a 7 Hz center frequency LFP oscillation (black line schematic of full 0–360° cycle) (top). The same neuron’s spiking binned by 7 Hz center frequency phase with SFC defined by the difference in spiking between the highest and lowest phase bins (bottom). **b**, SFC across neurons for a range of frequencies (~1–40 Hz). Neurons are sorted based on whether they were modulated by IEDs (as in Fig. 2a). **c**, SFC for IED-modulated neurons based on their modulation component firing pattern (as in Fig. 2e). **d**, Mean SFC in the 1–10 Hz bands across IED-modulated and non-IED-modulated neurons (****P = 3.1 × 10^−29^; *Z* = 11.2; 626,513 neurons; two-sided Wilcoxon rank-sum test). **e**, Mean SFC in the 1–10-Hz bands across component firing patterns (***P = 4.6 × 10^−9^, ***P = 3.0 × 10^−7^; *Z* = 5.9, 5.1; 233, 172 and 107 neurons; two-sided Wilcoxon rank-sum test). **f**, Higher SFC in the 1–10 Hz range at superficial depths (*P* = 2.0 × 10^−7^, *P* = 6.4 × 10^−4^ and *P* = 0.78; *r* = −0.33, −0.24 and −0.025; Spearman correlation for early-activation, suppression and late-activation neurons, respectively). Source data

**Extended Data Fig. 9:**
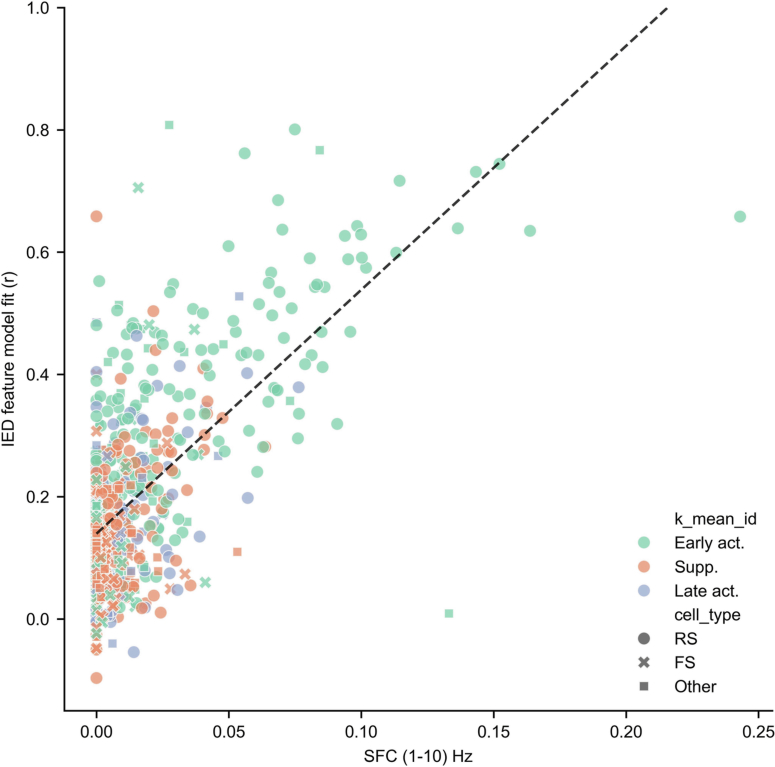
Correlation between amplitude coding and spike-field coherence. The relationship between spike-field coherence (SFC, as in Fig. 4) and IED amplitude coding (as in Fig. 3) is visualized (P = 5.2e-38, r = 0.46; Two-sided Spearman correlation). Neurons are colored by their component firing pattern. Source data

IEDs often occur in tissue that has baseline cognitive function, and so we hypothesized that IEDs are generated by neurons that, at baseline, participate in ongoing neurophysiological function^29^. Under this hypothesis, IEDs acutely commandeer (co-opt) these neurons and microcircuits, disorganizing ongoing cognitive processing and providing a possible mechanism for transient cognitive impairments (TCIs) in epilepsy shown in macroelectrode studies^25–28^. We first tested this hypothesis by examining whether neurons modulated during IEDs also demonstrated intrinsic coupling to local network oscillations, known to be important coordinators of physiological activity^55–58^. To the first point, we computed the spike-field-coherence of each neuron in our dataset to low-frequency oscillations of the micro-LFP (Fig. 4a). Neurons modulated during IEDs had higher overall spike-field coherence (SFC) (Fig. 4b) and this effect was most pronounced in early activation neurons (Fig. 4c). Indeed, neurons modulated by IEDs had significantly higher peak SFC in the 1–10 Hz range (Fig. 4d; *P* < 0.0001; two-sided Wilcoxon rank-sum test) and early activation neurons had significantly higher SFC than late-activation or suppression neurons (Fig. 4e; *P* < 0.0001; two-sided Wilcoxon rank-sum tests). Relatedly, neurons with the highest SFC generally localized to superficial neocortex (Fig. 4f; early activation: *P* < 0.0001, *r* = −0.33; suppression: *P* < 0.0001, *r* = −0.24; late activation: *P* > 0.05, *r* = −0.025; Spearman correlation of SFC and depth) and higher SFC correlated with a stronger coding of IED amplitude (as in Fig. 3i and Extended Data Fig. 9). Overall, this suggests that IEDs co-opt intrinsic physiological mechanisms of coordination, especially via regular-spiking early activation neurons that initiate and code the amplitude of the sharp discharge^48^.

**Fig. 5:**
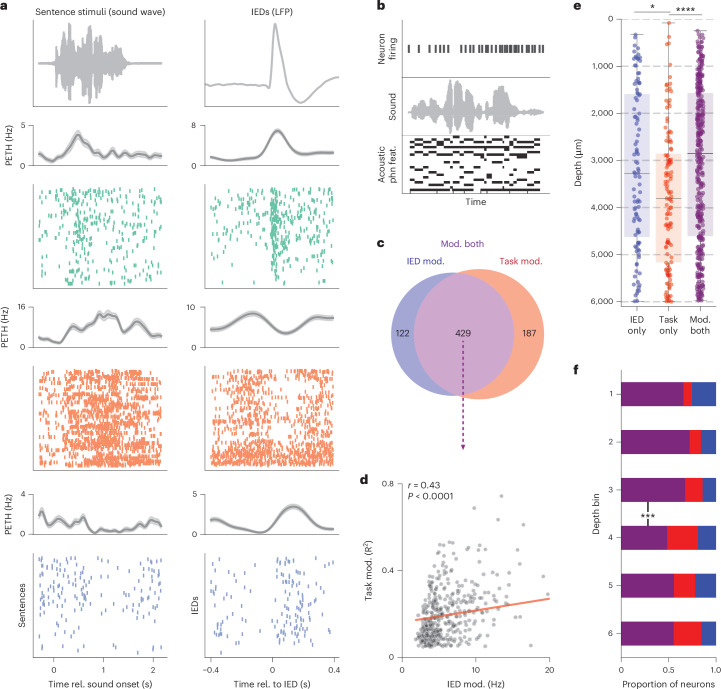
Shared neuronal substrates for IEDs and cognition. **a**, Firing from three example neurons, from each firing pattern as in Fig. 2e, aligned to onset of perceived sentences and IEDs. PETH overlaid, shaded error bars represent s.e.m. **b**, Schematic of the encoding model fit to measure perception task modulation. Models were fit to predict neuron firing from a set of acoustic-phonetic features derived from the presented sentences. **c**, Number of neurons across cortical insertion sites that are only modulated during IEDs (blue), modulated during IEDs and the perception task (purple; encoding model *r*^2^ > 0.05), or only modulated by the perception task (red). **d**, Within the set of neurons modulated during IEDs and the perception task, task encoding was correlated with IED modulation, as measured by the *r*^2^ and modulation depth (max–min firing rate) for perception and IEDs, respectively (*P* = 1.2 × 10^−20^; *r* = 0.43; two-sided Spearman correlation). **e**, Distribution of each group in *c* across neocortical depth (dotted lines: six discrete depth bins; *P = 0.012, ****P = 2.7 × 10^−5^; *Z* = 2.5, 4.2; 104, 120 and 348 for IED only/Task only/Mod. both neurons; two-sided Wilcoxon rank-sum test). **f**, Distribution of each group in *c* within the six discrete depth bins, showing highest concentration of shared neurons in the most superficial bins (***P = 0.0067; *Z* = 2.71; two-sided proportion *z*-test between proportion of ‘Mod. both’ neurons in bins 3 and 4). Source data

Building on this result, we next directly asked whether neurons modulated during IEDs also encoded meaningful information during ongoing cognitive processing. Three patients’ recording sessions occurred concurrently with a speech perception task (listening to naturalistic sentences)^59^. We observed individual neurons dually modulated by IEDs and sentence stimuli, (examples in Fig. 5a). Beyond modulation, we tested whether neurons encoded specific information about the acoustic-phonetic features of the stimuli (Fig. 5b and Methods). The majority of neurons significantly modulated by IEDs also encoded specific acoustic-phonetic features in the task (‘shared neurons’; Fig. 5c). Furthermore, the extent of speech encoding for a neuron was correlated with its modulation during IEDs, in other words neurons more strongly modulated by IEDs had stronger cognitive representations at baseline (Fig. 5d; Pearson correlation; *r* = 0.43, *P* < 0.0001). Shared neurons spanned neocortical depth, yet were more concentrated to superficial depths relative to speech-only neurons (Fig. 5e,f; *P* < 0.001, *P* < 0.05; Wilcoxon rank-sum test). This further suggests that the superficial neocortex is most strongly co-opted by IEDs. It also provides a potential basis for TCI as most neurons encoding cognitive information in our dataset were acutely co-opted when spontaneous IEDs occurred. Indeed, we found a striking distributional overlap of neurons modulated during IEDs and neurons that encoded cognitive information.

TCI^27,54,60^ and disorganization of ongoing computations was apparent in our dataset in one patient who had a measurable behavioral output of reaction time using a verbal semantic search task implicating the lateral temporal cortex. For trials in which an IED occurred either during the stimulus word or in the 1.5-s period immediately preceding it, reaction time was significantly longer than trials without an IED in this window (*P* < 0.05, *Z* = 612; Wilcoxon rank-sum test; *n* = 10, 90 IED and non-IED trials), mirroring previous work on lateral temporal IEDs^26^ and aligning with TCI literature from macroelectrode studies.

### Antecedent IED prediction

If IEDs could be predicted before becoming evident on macroelectrodes, new translational applications could include mitigating IEDs with neuromodulation to influence long-term excitability^6,7^ or reduce impacts on cognition^5^.

**Fig. 6:**
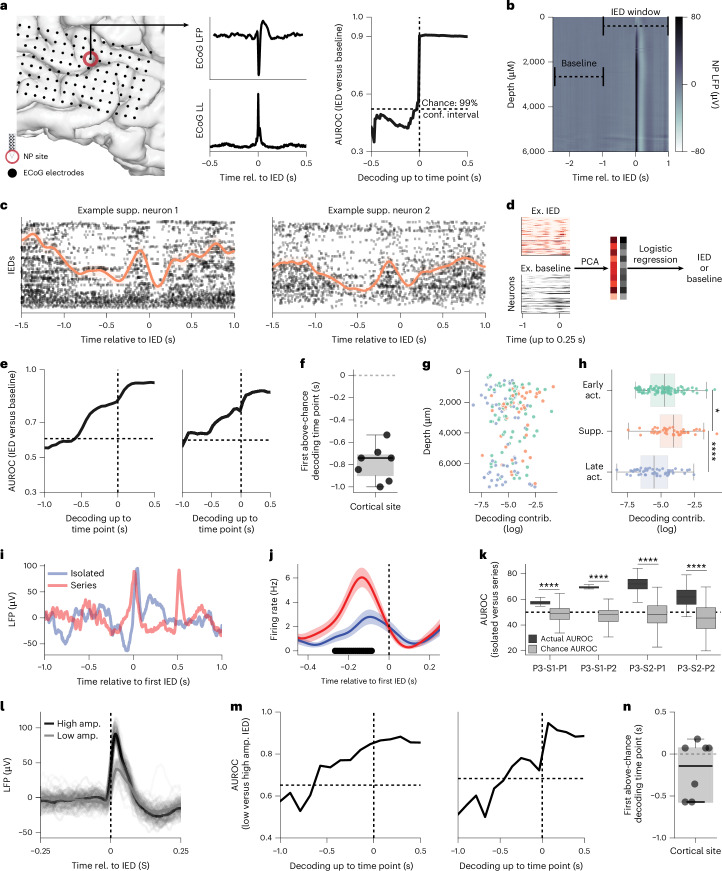
Predicting IEDs and their pathological features from single-neuron activity. **a**, Preoperative ECoG with the subsequent intraoperative NP site for an example patient. LFP (top) and instantaneous line length (LL) (bottom) averaged across IEDs at the ECoG electrode closest to the NP insertion site (middle). Predictability of IEDs versus pre-IED baseline windows (dotted line, 99% CI of AUROC from shuffle test) (right). **b**, Baseline and IED-forecasting windows, aligned to NP IED maximal slope. **c**, Raster plots of two suppression neurons that are modulated a second before IEDs. **d**, Model to predict IEDs at each time point. **e**, Predictability of IEDs in two sample sites. Horizontal line, 99% CI of chance prediction. **f**–**h**, The first above-chance decoding time point across sites (*n* = 7; Methods) (**f**). Neuronal contribution to forecasting IEDs across neocortical depth (**g**) and component firing pattern (*P = 0.012, ****P = 1.1 × 10^−5^; *Z* = 2.5, 4.4; 78, 46 and 54 neurons; two-sided Wilcoxon rank-sum test) (**h**). **i**, Example of isolated IEDs or IEDs that occur as a series. **j**, Example neuron with higher firing at the first IED of a series versus isolated event (dots; *P* < 0.05; two-sided cluster permutation test with multiple comparison correction). **k**, Decoding of isolated versus series IEDs based on neuronal firing from the −0.5 to +0.2-s interval around the initial IED. Chance distributions from shuffle test (****P = 1.0 × 10^−23^, ***P = 2.5 × 10^−34^, ****P = 3.5 × 10^−32^ and ****P = 1.5 × 10^−21^; *Z* = 10.0, 12.2, 11.8 and 9.5; 100 true and 100 chance; two-sided Wilcoxon rank-sum test). **l**, High- and low-amplitude IEDs (top/bottom quartile of IED amplitudes) for an example site. **m**, Predictability of low- and high-amplitude IEDs in two sample sites. **n**, The first above-chance decoding time point across cortical sites (*n* = 7) for predicting low- and high-amplitude IEDs. Source data

As an initial baseline for IED predictability with current technology, we annotated IEDs on preoperative electrocorticography (ECoG) recordings in three of our four participants (Fig. 6a). We then analyzed the LFP at the ECoG electrode under which the NP probe was inserted. To achieve similarity with previous IED detection algorithms and approximate real-time on-device detectors^9,42,61^, we took the instantaneous line length (calculated as the absolute value of the signal derivative) of the LFP (Fig. 6a). This transform functions effectively as a high-pass filter that accentuates epileptiform features and preserves temporal resolution^61,62^. We then used a logistic regression classification model, over time (Methods), to predict whether line length data were drawn from a baseline window or window immediately before the IED (Fig. 6a). This model could only distinguish the impending IED window from baseline immediately around IED maximal slope (Fig. 6a; −58.8–68.6 ms range across patients’ electrodes, −1.96 ms average decoding time relative to maximal slope). We next repeated this process with line length derived from the LFP of NP probes (Fig. 6b and Methods), achieving slightly improved predictive performance (−420.0 to +20.0 ms range across NP recording blocks, −164.0 ms average decoding time relative to maximal slope); however, performance was still limited. While line length is not a comprehensive feature of the LFP, its wide use as a biomarker in epilepsy neuromodulation is partially based on ease of approximation on fast timescales, in contrast to low-frequency oscillations. Equivalently, neuronal firing patterns are feasible to compute on fast timescales and have been used to drive numerous real-time brain–computer interface applications, including ambulatory platforms^33,63–65^.

As many neurons showed antecedent firing patterns much earlier than IED waveforms appeared on the LFP (Figs. 3a and 6c), we next assessed whether and how early IEDs could be forecasted with neuronal firing rates. We concatenated firing rates (downsampled to 20 Hz) across neurons for time points up to and including *t* (*t* = 0 is the time point of the maximal absolute IED slope). We used principal-component analysis (PCA) to extract a low-dimensional feature vector, capturing variance across time points and neurons (Fig. 6d). A logistic regression model decoded whether each trial corresponded to a baseline or peri-IED window. The AUROC at each time point was computed only using information up to and including that time point. Notably, in seven of nine cortical insertion sites, using enough temporally persistent neurons for model convergence (Methods), we could predict IEDs between 500 to 1,000 ms before the maximal slope (Fig. 6e,f; −1,000 to −534 ms range, −786 ms average). Suppression neurons contributed most to the decoder’s ability to predict IEDs (Methods) and contributions were distributed across depth (Fig. 6g,h; *P* < 0.05, *P* < 0.0001; two-sided Wilcoxon rank-sum test)

**Extended Data Fig. 10:**
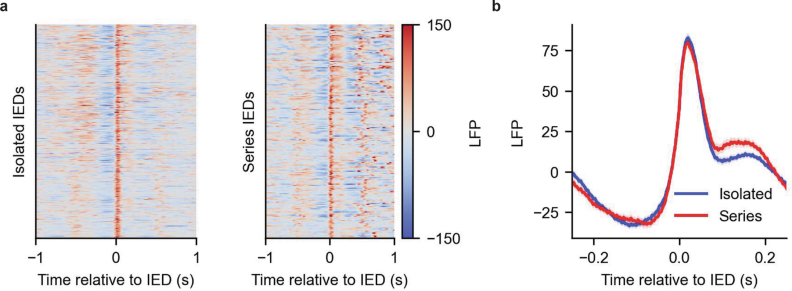
LFP characteristics of series and isolated IED. (**a**) Shown are heat maps of all isolated and series IEDs analyzed in Fig. 6. Series IEDs have additional peak(s) in voltage within the second following the first IED. In contrast Isolated IEDs return to baseline following the discharge. (**b**) The average LFP during Isolated and Series IEDs, centered on the discharge, showing minimal difference in peak amplitude between the two conditions. Source data

The propensity of IEDs to occur in short succession (as a series of discharges) or with higher amplitudes, can correlate with degree of cognitive impairment^51^ and seizure risk^66,67^. Predicting these features may thus have future implications for neuromodulation. We defined series IEDs as those that occur within a second after the previous IED (Fig. 6i and Extended Data Fig. 10) and we aligned neuronal firing to the onset of isolated versus series IEDs. Certain neurons increased IED antecedent firing rates more strongly if the IED would turn into a series (Fig. 6j), evident from −500 to +200 ms around the maximal slope of the first IED. From these modulations in firing rates, we could predict whether an IED would remain an isolated event or turn into a series, from the −500–200-ms window around the maximal slope of the first IED (Fig. 6k; *P* < 0.0001; two-sided Wilcoxon rank-sum test).

Finally, we performed the same predictive approach (Fig. 6e) with models trained to predict whether an upcoming IED would be low or high amplitude (bottom versus top amplitude quartiles; Fig. 6l). This was achievable up to 600 ms before the maximal slope in some insertions (Fig. 6m), yet the majority were closer to the IED maximal slope (Fig. 6n).

## Discussion

We recorded large-scale single-neuron firing across neocortical depth in awake, behaving patients with epilepsy, revealing complex yet predictable human cellular–laminar microcircuits for IED generation. In contrast to previous models that propose that IEDs form when hundreds to thousands of local neurons are drawn into a temporally synchronized burst^14–16,23^, we found that individual neuron firing patterns are asynchronous and span a much wider, nearly 2-s, timeframe before, during and after IEDs (the peri-IED window). This dynamic circuit involved apparent imbalances in excitatory and inhibitory dynamics across the cortical lamina, suggesting that IEDs are not random, unpredictable events, but might be anticipated up to 1,000 ms before their onset when tracked at the granularity of single neurons.

Neurons modulated during IEDs spanned the full depth of neocortical layers (Fig. 2j–m and Extended Data Fig. 1), had different action potential waveform types, and clustered into three groups based on their firing patterns. Together, these properties formed local microcircuits for IED generation. Firing dynamics revealed that even at the moment of the strongest IED deflection, firing was not abrupt and synchronous among neurons^14–16^. Instead, reliable cellular–laminar firing dynamics tiled the peri-IED interval, and even neurons within the early-activation group had nonzero cross-correlations with each other. Of particular importance were superficial regular-spiking early-activation neurons that coded the amplitude of the sharp IED discharge, fired with physiological oscillations and encoded information during normal cognitive processing. Putative inhibitory (fast-spiking) suppression neurons were modulated far before (1,000 ms) the discharge, indicating a decay of inhibition, and were most important for forecasting IED occurrence. These neurons were distributed across lamina and localized deeper relative to early-activation neurons. Finally, late-activation neurons were important for dictating the extent of the aftergoing slow wave of the IED.

A cortical microcircuit orchestrating recruitment of distinct cell groups during the peri-IED window could have clinical implications for new neurostimulation-based treatments. At present, closed-loop stimulation for epilepsy (RNS) remains a ‘reactive treatment’ in which electrical current is delivered to the brain directly after epileptiform waveforms are detected in local macro-contact signals (note, despite the historical intention and ability of RNS being to abort acute ictal events, most of the closed-loop stimulation deliveries each day in RNS are in response to interictal events that would not become seizures)^68^. Excitatory–inhibitory imbalances enabled prediction of upcoming IEDs up to 1,000 ms before IED deflection, opening doors for preemptive stimulation. Such ‘proactive stimulation’ before an IED fully forms might enable a more effective means of tempering epileptiform activity through neuroplastic changes in the seizure onset network over time, and mitigating immediate pathological effects of IEDs^69^ on cognition^70^. This has important clinical implications given that, currently, less than 20% of patients achieve seizure freedom, on the timeframe of years, with RNS. These points are key implications of this work but would require proper randomized controlled trials in the future.

Leveraging NP to simultaneously record from far more neurons than most previous approaches^17,29^ enabled not only single-site IED prediction analyses, but also the mechanistic study of IED associations to transient cognitive impairments^5^. The majority of neurons linked to IEDs also encoded specific perceptual information during cognitive processing (language task; Fig. 5c). Relatedly, the firing patterns of many of these neurons were paced by physiological neural rhythms (local LFP oscillations) as revealed by SFC (Fig. 4). These results align with the anatomical locations of these neurons in the superior and middle temporal gyri, areas implicated in IED-related transient cognitive impairment during language processing^26^. The overlap of IED-modulated neurons and neurons that encode cognitive information provides a potential cellular basis for TCI and confirms hypotheses that IEDs leverage neurons that, at baseline, are important for ongoing cognition. Future work will clarify whether the high overlap of neurons involved in both IEDs and cognition is due to preferential engagement of units involved in cognition (for example due to intrinsic circuit characteristics), or indiscriminate local incorporation into IED microcircuits.

Together, our results (illustrated in Fig. 5) suggest that when neurons engaged in neural rhythms and/or dynamic processing of a cognitive task are suddenly drawn into IED generation, their firing related to computations and/or information representations (for example rate coding) is vulnerable to temporary disruption. This potential mechanism further motivates suppression of IEDs as a direct treatment for pervasive cognitive impairments in epilepsy^4,26,71–74^. Our work also implies that stimulating in specific neocortical depths might enable further microscale optimizations for closed-loop systems (for example superficial layers, containing more early-activation neurons co-linked to IED features and physiological oscillations; Figs. 3i and 4g).

High-density probes facilitate the recording and isolation of higher numbers of neurons than microwire approaches^75^. The number of cells per electrode, however, may seem lower than traditional microwires, due to the limited extent of coverage (single NP insertion), intraoperative recording challenges (motion and electrical artifacts) and that SOZ tissue may have fewer units relative to healthy tissue. Our cell yield is on par or improved relative to other human studies using NP in vivo^35,76,77^, and active efforts are underway to improve recording techniques and neuronal yield in future work^36,76^.

The differentiation of putative neuron types has been another major challenge in animal and human single-unit recordings. Multichannel high-density recordings can improve this differentiation^37,78^. The shape and polarity of recorded waveforms relate largely to cellular morphology differences in regular-spiking cells (putative pyramidal cells, which have a somato–dendritic axis) and fast-spiking cells (putative interneurons, which typically have a more radial orientation of dendrites in many directions), as well as the relative recording location along these cells^79^. Based on NP orientation, we would expect the transmembrane currents of pyramidal cells to therefore be transduced into negative waveforms on Neuropixel recording electrodes near the soma (regular-spiking waveform) or a positive waveform from axonal spikes (positive-spiking waveform, see Jia et al.^37^ and Someck et al.^78^). By contrast, interneurons would be less prone to waveform differences from different recording surface locations, with a relatively consistent narrow monophasic negative waveform morphology. Multichannel spatiotemporal analysis showed robust differentiation of these subtypes in this study^37^^,^^38^ (Extended Data Fig. 2).

Limitations of this study include the cohort size, which was primarily due to the rarity of human intraoperative NP recordings coupled with the uncertainty of recording sufficient IEDs in a given tissue site (IEDs may not be observed even if recording in the SOZ). Relatedly, given technical limitations of awake human NP recordings, our sampling was limited to epileptogenic zones in the lateral temporal neocortex, motivating future studies of additional brain areas such as the hippocampus and amygdala. While IEDs were present in the NP LFP waveforms engaging local neuronal populations, we cannot confirm the precise origin locations and whether IEDs were locally generated or propagated to the NP site; however, our CSD analyses were concordant with locally generated IEDs^22,23^, aligning with many neurons being modulated before (up to 1,000 ms before) the IEDs. Additionally, all recording sites were within the pathological, epileptic network; however, future studies of simultaneous NP and high-density intracranial electroencephalography recordings across the cortex are needed to definitively confirm whether IEDs are locally generated or propagated to the NP site. While our CSD analysis helped to understand the relative depth positions of sinks and sources, precise cell layer locations should be the focus of future investigations, as it was not definitively possible due to operative and histological constraints.

This study takes key steps forward in understanding the mechanisms of human IEDs at the single-neuron level and the neuronal–laminar interactions of cortical sites that give rise to epileptiform activity. Namely, rather than neurons stochastically being drawn into synchronous firing, IEDs are born out of structured microcircuits that span cortical depth (Fig. 3a–e) and leverage asynchronous patterns of neuronal firing. Pathological IEDs features, such as the discharge amplitude and whether an IED will remain isolated or progress to a series, are shaped through firing of distinct neurons within the overall microcircuit. Superficial regular-spiking neurons were important in coding the amplitude of the sharp discharge, whereas fast-spiking neurons were modulated far before the discharge and were important for forecasting IEDs. We also demonstrate that the pathophysiology of IEDs is rooted in their co-opting of single neurons that code cognitive information and adhere to physiologic oscillations. This confirms previous speculation of cognitive impairment (TCI) mechanisms, namely that IEDs induce TCI by diverting underlying neurons out of cognitive processing.

Overall, the use of NP probes during awake epilepsy surgery facilitated a previously unobserved lens into the highly structured laminar basis of IEDs, as well as the interplay between IEDs and normal cognitive physiology. In addition to these mechanistic insights, single-neuron activity could predict not only IEDs, but also their pathological features. Leveraging such approaches, in line with rapid advances in single-neuron neurostimulation devices^80–82^, may enable future therapeutic devices that predict and mitigate IEDs before they occur as a new avenue for ameliorating cognitive impairment and seizures for people with epilepsy.

## Methods

### Participants

We evaluated 11 patients with epilepsy undergoing resective surgery for treatment of seizures, across which 24 probe insertions were screened for IEDs. Overall, nine probe insertions had at least 50 IEDs, which was deemed sufficient for this study, and these patients were included in this study. These recordings came from four patients (three female and one male, aged 42, 37, 31 and 31 years). Probes were inserted in cortical tissue that was then resected for surgical treatment of seizures related to epilepsy or neoplasm. Participants provided informed, written consent before the surgery for NP probes to be placed temporarily during the procedure. Study protocols were reviewed and approved by the University of California, San Francisco Institutional Review Board (IRB).

Before the resective surgery above, three of the four patients also underwent inpatient electrocorticography recordings using subdural and depth electrode arrays to help localize the SOZ for surgical planning. Voltage signals of this neural data were recorded via a multichannel amplifier that was optically connected to a digital signal processor (Tucker-Davis Technologies). Signals from each contact were amplified and recorded at a sampling rate of 3,052 Hz for brief recording sessions that were roughly 5–10 min in length. One representative session was chosen for each patient for analyses that compared IEDs recorded with ECoG to IEDs recorded with NP probes, as described below.

### Neuropixels array and placement

In general, NP probe placement followed our previous work^36,39^. A detailed description of the hardware configuration can be found in Chung et al.^36^. In brief, the hardware configuration consisted of a NP 1.0 NHP short probe (10-mm-long shanks) with appropriate headstage and cables. The NP probe was attached to a custom designed probe stabilizer and mounted on a micromanipulator (Narishige M-3333) attached to a three-joint mounting arm (Noga NF9038CA) and clamp (Manfrotto 386BC-1). Components were tested preoperatively and ethylene oxide sterilized with IRB-approved cycle parameters. For two patients, a double-probe apparatus was used to simultaneously record from two distinct cortical sites. The double-probe apparatus consisted of two linearly arranged probes, 2 mm apart.

NP probe placement was determined after clinical mapping and the resection zone was defined. In all recordings, probes were inserted into tissue that was subsequently resected. Once a site was identified, the micromanipulator lowered the NP probe through an aperture in the probe stabilizer at 50–75 μm s^−1^. A final depth of approximately 6–8 mm from the brain surface was targeted at a trajectory perpendicular to the targeted gyrus. Care was taken to ensure a portion of electrodes remained above the cortical surface to aid in later annotation of the cortical surface and each isolated neuron’s depth. Neural signals from the NP probe were acquired using standard acquisition systems^83^ and SpikeGLX software. Neural signals were referenced using a monopolar montage to a subgaleal reference contact inserted in the scalp.

### Neuropixel recording sessions

All participants were awake during recording and three participated in a task with passive listening to natural speech sentence samples^59^. Recording sessions typically lasted for at least several minutes with a range of continuous usable (artifact-free) data approximately 520–1,380 s in durations across patients, and three of four patients had recordings in multiple distinct sites (Supplementary Table 1).

### Isolation of IEDs

The voltage data from the ECoG and NP LFP recordings herein were interpreted and annotated by a board-certified epileptologist (J.K.K.), blinded to all single-unit data, using a semi-supervised approach^62^. Recordings were visually examined and time periods containing signal artifacts (noise) were first excluded from analysis to avoid detection contamination from data skewing. The onset and offset of individual IEDs were detected by first using an LL-based automated detector using a 40-ms window (https://github.com/Kleen-Lab/Linelength-spike-detector-MATLAB). This was followed by manual adjustment of screening and correction for false positives and negatives (MATLAB). IEDs consisted of single interictal spikes, sharp waves or bursts of epileptiform activity identified as waveform deflections from baseline with or without aftergoing slow waves and/or overlying pathological fast frequency activity. Each IED within a repetitive spike run was marked individually. We avoided annotating electrical field deflections that appeared due to artifact or volume conduction only. In the case of double-probe recordings, the timing and morphology of IEDs on the two probes was indistinguishable, so annotated IEDs were extrapolated from one of the probes to the other. Following annotation steps, the time point of maximal absolute voltage deflection (maximal absolute slope) within the annotated onset and offset duration was centered for each IED and used for temporal alignment (time zero) in all subsequent analyses.

### Current source density analysis of IEDs

We performed CSD analysis on each recording aligned to the onset of IEDs. We first smoothed the LFP with a Gaussian kernel of standard deviation 15 electrodes in the spatial dimension and 11.7 ms (six samples) in the time dimension. CSD was then computed as the second spatial derivative at each time point across IEDs. To assess significance, we randomly selected the same number of time points, as IEDs, for each block 200 times and computed the null-aligned CSD. Significant values were required to be greater than the 99.5th percentile of the null CSD distribution.

### Isolation of single-neuron spiking

To isolate putative single-neuron activity (action potential neuronal spikes or ‘spiking’; not to be confused with IEDs), NP recordings were high-pass filtered, with a cutoff of 300 Hz at rate of 30 kHz, and spike-sorted using Kilosort 2.5 with standard parameters^84^. Post hoc motion correction was applied using Kilosort 2.5 and, for one patient, manual trace-based registration. Clusters identified with Kilosort 2.5 were manually curated using a custom graphical user interface (MATLAB 2023; https://github.com/yaxigeigei/MTracer.git). Clusters with excessive refractory period violations, nonphysiological waveforms or multiple nonseparable waveforms were discarded. The remaining clusters were subsequently analyzed as putative single neurons. To quantify the depth of each neuron in the neocortex, we first isolated which electrode on the NP array corresponded to the cortical surface. This was carried out by visually inspecting the LFP and identifying the NP electrodes that were not inserted into the neocortex. A characteristic LFP pattern can be seen at the transition between cortical tissue and extracortical saline (the tissue–saline interface; Supplementary Fig. 1). Depth was then defined as the relative distance (μm) of each neuron along the probe minus the probe depth at the cortical surface.

Spike waveform analyses were conducted by averaging the high-pass filtered signal at spike times for each neuron on the ten closest electrode contacts^39^. This provided a spatiotemporal waveform for each neuron, consisting of a spike averaged high-pass-filtered signal centered on the closest channel to the neuron with a total of ten channels considered (Extended Data Fig. 2). We then embedded the spectrotemporal waveform across neurons into a low-dimensional space using UMAP. Three clusters were clearly visible and can be defined with a simple linear Gaussian mixture model. We also computed the trough-peak time for each fast-spiking and regular-spiking neuron, defined as the time difference from the absolute minimum to the subsequent local maximum on the waveform of the closest electrode to the neuron.

Binned firing rates (also referred to as peri-event time histograms; PETHs) for each neuron were computed by counting the spike events in 100-ms sliding windows (overlapping bins) with step size of 10 ms. PETHs were smoothed with a Gaussian kernel (40 ms std.) Given the technical limitations of human intraoperative NP recordings^35,36^, neuronal drift is common due to movement during recording blocks. Practically, this meant that some neurons were captured only for parts of the recording block. To prevent this bias (for example, artificially low firing rates in PETHs), when calculating the average IED-aligned PETH for each neuron we only considered peri-IED intervals where the neuron had at least a single action potential (neuronal spike). This ensured we only analyzed periods in which a given neuron was effectively sampled during the block (see section ‘Single-neuron activity during IEDs’), leaving the dynamic timing of its firing as the central focus of analysis.

### Analysis of neuronal firing aligned to IEDs

To identify any low-dimensional patterns in firing aligned to IEDs across all recorded neurons (Fig. 2), we first aligned neuronal PETHs to a peri-IED window of −400 to 400 ms around the IED maximal absolute slope. We then *z*-scored each PETH across time over its peri-IED time interval and then applied *k*-means clustering across neurons to discover three template firing patterns during the peri-IED window^39^. Three clusters were chosen based on the elbow method of added variance^85^. To assess whether fast-spiking neurons were significantly enriched in the suppression component, we bootstrapped a 95% confidence interval for the proportion of fast-spiking cells in each *k*-means component. In brief, we sampled 2,000 times, with replacement, cells within each *k*-means component and calculated the proportion of those cells that were fast-spiking-positive in each iteration. We then calculated a 95% confidence interval for the proportion of fast-spiking-positive cells from this bootstrapped distribution. The confidence interval for *k*-means component 2 (suppression) did not overlap with that of components 1 or 3. Of note, this analysis was independent and not informed by the UMAP clustering performed on the LFP during IEDs (Extended Data Fig. 1).

### Cross-correlations across circuit elements

To assess functional connectivity between neurons we computed cross correlograms between neuronal spiking within a NP insertion. For this analysis, we utilized only those neurons that were strongly modulated during IEDs (*P* < 0.0001; a subset of ‘modulated’ neurons in Fig. 2a). In each NP insertion, we computed the pairwise cross correlogram between neurons that met this criteria. We used a bin size of 5 ms and a maximal correlation lag of ±500 ms. To assess statistical significance of the cross-correlation for each pair of neurons, we applied a permutation test. In each of 100 iterations, we randomly shuffled the spike times for each of the two neurons and computed a null cross-correlation from which we took the maximum value across lags. The result was a 100-dimensional null distribution of maximum correlation values. Neuron pairs where the maximum of the true cross correlogram exceeded the 97.5th percentile of the null distribution were considered significant. We then averaged significant cross correlograms, across pairs of neurons, based on their peri-IED response type (Fig. 2e).

### Assessing single-neuron coding of IED features

We fit a time-lagged linear model to predict each modulated neuron’s PETH from specific features of the IED by first averaging the micro-LFP across NP electrode contacts to yield a single time series. For each IED, we extracted the amplitude and time point corresponding to the overall, signed maximal amplitude, minimum amplitude before the discharge, and minimum amplitude after the discharge. We normalized these features to between 0 and 1, then fit a ridge regression temporal receptive field model (TRF)^86^ with L2 regularization and receptive window of ±350 ms relative to the input features. In brief, this model learns a set of weights that best predict each neuron’s PETH, having dimensions *T* × *F*, where *T* is 70 (number of time points in the 700 ms window at 100 Hz) and *F* is 3 (number of IED features). We searched a logarithmically spaced range of L2 regularization parameters from 0.1 to 100. Models were fit on 90% of the data and evaluated on held-out 10%. To evaluate performance, a linear correlation value, *r*, was computed between predicted and ground-truth PETHs on the held-out data. We considered neurons with *R*^2^ values that exceeded 0.05 (ref. ^87^) for subsequent analysis. Maximal tuning to each feature was assessed by taking the maximum absolute value across time for each feature’s coefficient vector. To better understand how the aftergoing slow wave, and preceding slow changes, were generated, we computed the average neuronal firing across the three neuron types (early activation, late activation and suppression) in the windows of −0.25 to −0.05 and 0.15–0.25 around IED onset across IEDs. We also computed the average LFP in these same windows across IEDs. We then performed regression to relate mean activity across each of the three neuron types with the LFP magnitude of the antecedent and aftergoing slow wave. We also computed the LFP-absolute-slope-triggered average for each neuron. To do this, we first took the instantaneous derivative of the LFP. For each neuron we then computed an evoked response potential of the LFP absolute slope aligned to the neuron’s spikes (spike-triggered average of the LFP absolute slope).

### Spike-field coherence

To relate the neuronal spiking directly to the underlying LFP signal, we assessed the SFC of all neurons across cortical insertion sites. This was performed over the entire recording blocks, excluding peri-IED time points to mitigate artifacts in phase calculation caused by the sharp IED deflection. For each neuron we calculated a histogram of spike count based on the instantaneous phase of low-frequency oscillations (Fig. 4a). We evaluated neurons that had at least 100 spikes in their respective recording, and decomposed the LFP into frequency bands. We notch-filtered the LFP at harmonics of 60 Hz and decomposed the LFP into a set of 40 frequency bands, spanning 1–50 Hz with base 2 logarithmic spacing. We applied Morlet wavelets (seven cycles^88^) to each center frequency and took the phase of the complex signal producing a time series of phase values, to which we aligned neuronal spiking via circular histogram of counts. We used the phase at the electrode closest to the median depth across the neuron’s spikes (the same channel from which the spike waveform was computed). We analyzed all neuronal spikes across the recording block except those within 0.5 s from the IED center points (maximal absolute slope as defined above) to mitigate artifacts in phase computation caused by the sharp IED deflection^62^. We discretized phase into ten equally spaced bins ranging from −3.14–3.14 radians (36° bins). SFC was defined as the difference in maximum and minimum proportion of spikes across the phase bins^89^ (Fig. 4a). For significance testing relative to chance, for each neuron we randomly shuffled the neuronal spike times 500 times and computed a null-phase histogram. Neurons that had significant SFC were defined by the true-phase histogram being smaller or larger than the 95% confidence interval of the null distribution for at least one phase bin.

### Neuronal encoding during speech perception

To test whether neurons that were modulated by IEDs were also modulated during a speech perception task, as predicted by previous work^25,26^ we leveraged portions of the recording sessions in which patients performed a naturalistic speech perception task. Specifically, three of the four patients passively listened to 110 unique sentences from the TIMIT speech corpus^59^, which spans a large linguistic feature space. Acoustic-phonetic features^90^ were hand-labeled for each sentence in the TIMIT corpus and aligned to neuronal firing time series. Similar to IED feature analysis, TRF models were fit to predict each neuron’s PETH from acoustic-phonetic features of the speech stimuli^39^. Here, we computed PETHs with a sliding 50-ms, rather than 100-ms window, given that acoustic features of speech evolve on a rapid timescale. Before TRF modeling, acoustic-phonetic features and PETHs were *z*-scored, aligned and resampled to 100 Hz. TRF models were similarly fit using cross-validated ridge regression on a training set comprising 100 sentences and performances were evaluated by computing the correlation (*r*) between ground truth and predicted PETHs for each neuron on a test set comprising 100 sentences (each of the ten remaining sentences repeated ten times). We further considered all neurons with an *R*^2^ above 0.05. The fourth patient performed a semantic association task (‘semantic search’) in which a single stimulus word was given and the participant was instructed to generate a word that fell into a similar or opposite semantic category. For example, if the word was ‘good’ the patient may respond with ‘fine’ or ‘bad’ based on whether they were prompted to generate a semantically similar or dissimilar word, respectively. Reaction time (duration from stimulus offset to speech onset) was measured as a behavioral output. IED trials were defined as those in which an IED occurred within 1.5 s before the offset of the stimulus prompt or before the participant repeating the target word.

### Antecedent IED prediction from neuronal ensembles

We designed models to predict IEDs in two scenarios: from single-neuron firing, and from the LFP of NP electrodes (line length derivation). As a control, we also trained and tested these models using the line length of ECoG electrodes that were placed over NP recording sites during presurgical monitoring. An instantaneous line length time series transformation was computed as the absolute value of the derivative of the LFP, as line length is a signal biomarker commonly utilized for the detection of epileptiform activity and RNS^9,61,62^.

To determine the extent to which IEDs were predictable using line length, a commonly used predictive feature in current macroelectrode IED and seizure detection algorithms^9,61^. For each recording block, we aligned the LFP to the maximal IED slope. We extracted two intervals: a baseline window (−2.5 to −1 s relative to maximal slope) and an IED-forecasting window (−1–0.5 s relative to maximal slope) (Fig. 6b). We next extracted the instantaneous line length from the LFP at each NP electrode contact. This can also be seen as an analogous single dimensional LFP-based feature to neuronal firing rates. The procedure described below was also used to predict IEDs from the LFP with line length used instead of single-neuron firing rates.

We aligned single-neuron firing to two intervals: a baseline window (−2.5 to −1 s relative to the IED maximal slope) and an IED-forecasting window (−1–0.5 s relative to IED maximal slope). Here, we filtered for neurons that were stable and captured for at least half of the overall number of IEDs in the recording block (seven out of nine columns had sufficient number of neurons). We next downsampled neuronal firing rates to 20 Hz. At each iterative time point (*t*) along the IED-forecasting and baseline windows, we concatenated the firing rate across neurons for time points up to and including time point *t*. PCA was then applied to extract a ten-dimensional feature vector, capturing variance across time points and neurons. We next trained a logistic regression model for each time point along the window to classify whether trials corresponded to the baseline or IED-forecasting window. This model was evaluated using leave-one-out cross validation to maximize the number of IEDs available for model training. The AUROC score at each time point then reflects how well neuronal firing up to and including that time point can be used to distinguish an upcoming IED from baseline activity. Null distributions were computed by shuffling the baseline and IED labels 300 times and computing the AUROC score. We took the 99th percentile of this distribution as a conservative estimate on the upper bound of chance decoding performance.

We next implemented the decoding analyses above to predict IED pathological features, specifically amplitude, and whether an initial IED would continue in a series of repetitive IEDs^51,91^. For predicting amplitude, the logistic regression model was trained to classify between high-amplitude and low-amplitude IEDs (rather than baseline versus peri-IED windows), defined as being in the top or bottom quartile of all IED amplitudes within the recording block, respectively. For predicting whether an IED would become a repetitive series of IEDs, we aligned neuronal firing to solitary IEDs and to the initial IED in any series. We defined the onset of a series IED to be the maximal slope of the first IED after which at least one more IED followed within 1 s. We defined the onset of an isolated IED as the maximal slope of an IED after which no subsequent IED occurred for at least 2.5 s, the length of the previously defined baseline window. Only a few cortical insertion sites (four of nine) had sufficient series IEDs (>25) to assess predictability. In these insertion sites, series of IEDs were less common than isolated IEDs, so we randomly sampled the isolated IEDs, without replacement, to match the number of series IEDs 100 times to ensure balanced classes for binary classification model training. During each iteration, we computed the receiver operating characteristic score using leave-one-out cross validation. To compare to chance performance, we repeated this process another 100 times and shuffled the labels for series and isolated IEDs each iteration. We then performed nonparametric Wilcoxon rank-sum tests to assess significant differences between the shuffled (null) and true AUROC distributions.

### Statistical analysis

Methods for assessing statistical significance are described in the main text and figure captions. In brief, we used nonparametric Wilcoxon rank-sum tests to assess significance between unpaired distributions and Wilcoxon signed-rank tests to assess significance between paired distributions. To assess significance of decoding performances and SFC, we created null distributions via iterative labeling shuffling as described above. Specific procedures and parameters for null distributions are specified elsewhere in the relevant sections. Boxplots across all figures and panels depict median (horizontal line inside box), 25th and 75th percentiles (box), 25th and 75th percentiles ±1.5× the interquartile range (whiskers) and outliers (dots). Across all figures and panels, shaded error bars reflect s.e.m. Code and data to replicate the main findings of the manuscript are available at https://github.com/asilvaalex4/NP_IEDs and 10.5281/zenodo.18651142 (ref. ^92^), respectively

### Reporting summary

Further information on research design is available in the Nature Portfolio Reporting Summary linked to this article.

## Online content

Any methods, additional references, Nature Portfolio reporting summaries, source data, extended data, supplementary information, acknowledgements, peer review information; details of author contributions and competing interests; and statements of data and code availability are available at 10.1038/s41593-026-02258-4.

## Supplementary information

Supplementary Information Supplementary Fig. 1 and Supplementary Table 1

Reporting Summary

Supplementary Data 1 Statistical source data for Supplementary Fig. 1.

## Source data

Source Data Fig. 1 Statistical Source Data.

Source Data Fig. 2 Statistical Source Data.

Source Data Fig. 3 Statistical Source Data.

Source Data Fig. 4 Statistical Source Data.

Source Data Fig. 5 Statistical Source Data.

Source Data Fig. 6 Statistical Source Data.

Source Data Extended Data Fig. 1 Statistical Source Data.

Source Data Extended Data Fig. 2 Statistical Source Data.

Source Data Extended Data Fig. 3 Statistical Source Data.

Source Data Extended Data Fig. 4 Statistical Source Data.

Source Data Extended Data Fig. 5 Statistical Source Data.

Source Data Extended Data Fig. 6 Statistical Source Data.

Source Data Extended Data Fig. 7 Statistical Source Data.

Source Data Extended Data Fig. 8 Statistical Source Data.

Source Data Extended Data Fig. 9 Statistical Source Data.

Source Data Extended Data Fig. 10 Statistical Source Data.

## Data Availability

Data to replicate the main findings and all figures of this manuscript are available at 10.5281/zenodo.18651142 (ref. ^92^). Given that participants at the onset of the study did not consent to public release of their data, raw data will be made available from the corresponding author (E.C.) upon request to protect patient privacy and consent. Source data are provided with this paper.

## Code availability

Code to replicate the main results and all figures of the manuscript is available at https://github.com/asilvaalex4/NP_IEDs. Code for IED detection via the line length algorithm is available at https://github.com/Kleen-Lab/Linelength-spike-detector-MATLAB. Code for spike sorting and cluster curation is available at https://github.com/yaxigeigei/MTracer.git.
