# Supplementary material for: Laminar organization of cellular microcircuits modulating human interictal epileptiform discharges
Source: Nat Neurosci. 2026 Apr 30;29(6):1462–75. doi: 10.1038/s41593-026-02258-4 (PMC13246451; doi:10.1038/s41593-026-02258-4)
Supplement: Supplementary file 1 — Supplementary Fig. 1 and Supplementary Table 1 [file 41593_2026_2258_MOESM1_ESM.pdf]

nature neuroscience

Supplementary information https://doi.org/10.1038/s41593-026-02258-4

Laminar organization of cellular microcircuits modulating human interictal epileptiform discharges

In the format provided by the authors and unedited

Contents:

Supplementary Figure 1

Supplementary Table 1

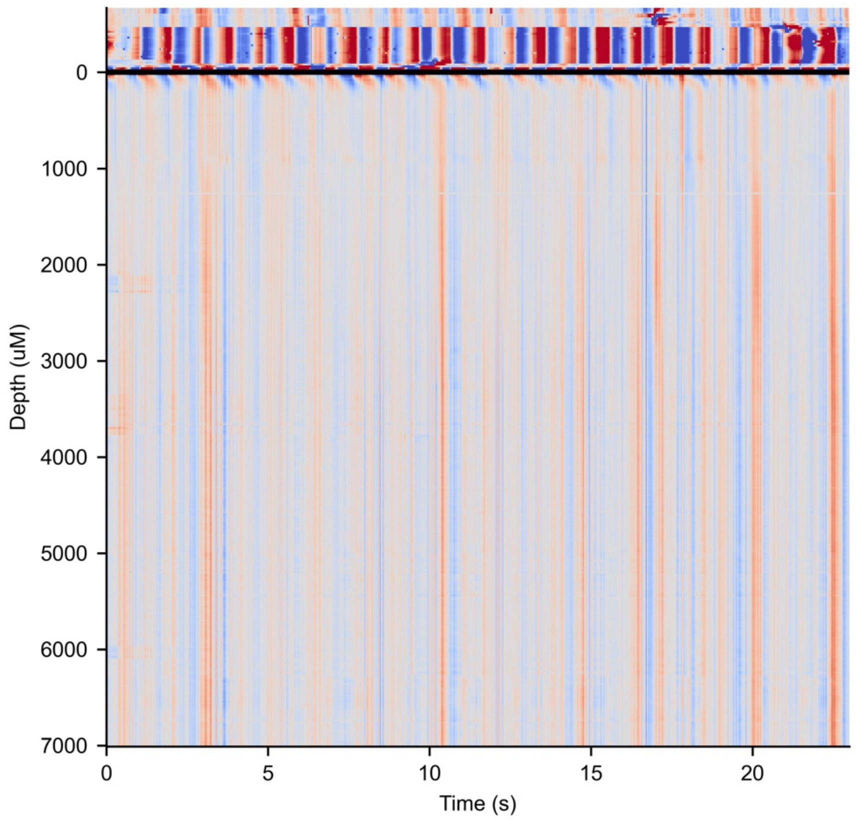

Supplementary Figure 1: Characteristic LFP signal at the pial surface. Shown is the LFP from a recording segment with P1-S1. Note the characteristic LFP signal at the pial-saline interface (marked 0 as the cortical surface). The distinct signals are due to different impedance of electrodes in air, saline, and brain tissue and are a reliable marker of the brain surface.

Pt	Site	# Probe	Region	Duration (s)	# IEDs	# Neurons	# RS	# FS	# PS	Epileptogenic zone	Age	Sex
1	1	1	mSTG	1045	101	131	37	53	41	Lateral and basal temporal cortex	42	F
	2	1	pMTG	520	184	14	0	9	5			
2	1	1	mSTG	605	171	170	78	27	65	Hippocampus, amygdala, and lateral temporal cortex	37	M
3	1	2	mSTG	970	309	94, 130	66, 42	9, 39	19, 49	Lateral and basal temporal cortex	31	F
	2	2	mSTG	1380	258	181, 189	129, 29	30, 101	22, 59			
4	1	2	aSTG	700	71	93, 150	75, 131	5, 6	13, 13	Anterior lateral temporal pole cortex	31	F

Supplementary Table 1: Neuropixels recordings included in analysis. Across four patients, data was collected from nine cortical insertion sites. The table specifies the sites, number of probes, region, duration (in seconds), number of IEDs, and neuron yields (RS, FS, PS neurons) from each recording in our patient cohort.
